# Concurrent Four‐Dimensional Dynamic MRA and Perfusion Imaging Using Dual‐Module Arterial Spin Labeling MRI With Stack‐Of‐Stars Golden‐Angle Radial Acquisition

**DOI:** 10.1002/nbm.70359

**Published:** 2026-07-22

**Authors:** Tianrui Zhao, Jianing Tang, Sarah J. Moum, Yining He, Ziyi Huang, Ayaz Khan, Ali Shaibani, Lirong Yan

**Affiliations:** ^1^ Department of Radiology, Feinberg School of Medicine Northwestern University Chicago Illinois USA; ^2^ Department of Biomedical Engineering Northwestern University Evanston Illinois USA; ^3^ Department of Medical Imaging, Ann & Robert H. Lurie Children's Hospital of Chicago Chicago Illinois USA

**Keywords:** arterial spin labeling (ASL), bSSFP, cerebral blood flow (CBF), dynamic MR angiography (MRA), golden‐angle radial, perfusion

## Abstract

Arterial spin labeling (ASL) has been used for perfusion imaging and non‐contrast enhanced dynamic MR angiography (MRA), and both have become favorable for clinical diagnosis and treatment planning of cerebrovascular diseases. Separate sequences are typically required to obtain brain vascular hemodynamics and downstream perfusion information. However, concurrent dynamic MRA and perfusion imaging within a single acquisition can provide spatially co‐registered macrovascular and microvascular maps to enhance diagnostic confidence and efficiency. In this work, we developed a dual‐module ASL technique to concurrently obtain four‐dimensional (4D) MRA and cerebral perfusion images from a single scan. Specifically, pseudo‐continuous ASL (pCASL) and pulsed ASL (PASL) modules were integrated and encoded with a 3D stack‐of‐stars golden‐angle radial acquisition. 4D MRA and perfusion contrasts were generated through pair‐wise subtractions. Sparsity‐constrained image reconstruction was used in generating 4D MRA and perfusion images from different portions of radial k‐space data. Both numerical simulationsL.  and in vivo experiments were performed to demonstrate technical feasibility. Both time‐resolved MRA with high spatiotemporal resolution and perfusion‐weighted images with good contrast were successfully obtained from a single scan using the proposed dual‐module ASL technique. The performance of the proposed technique was compared against the reference 4D MRA technique in terms of vascular delineation and blood flow dynamics, and the conventional pCASL perfusion imaging with 3D GRASE for cerebral blood flow (CBF) measurement. The dual‐module ASL showed comparable performance in depicting arterial blood flow dynamics and gray matter CBF quantification (*p* = 0.73) to the reference 4D MRA and 3D GRASE pCASL, respectively. These results indicate the feasibility of the proposed technique for concurrent 4D MRA and perfusion imaging from a single scan, which could be a potentially powerful imaging tool for the detailed characterization of dynamic blood flow patterns through the cerebrovascular structure and downstream perfusion.

## Graphical abstract

A dual‐module ASL technique is proposed to obtain both 4D MRA and cerebral perfusion images within a single scan. Both time‐resolved MRA with high spatiotemporal resolution and perfusion‐weighted images with good contrast were successfully obtained from a single scan using the proposed dual‐module ASL technique, suggesting that it could be a potentially powerful imaging tool for the detailed characterization of dynamic blood flow patterns through the cerebrovascular structure and downstream perfusion.

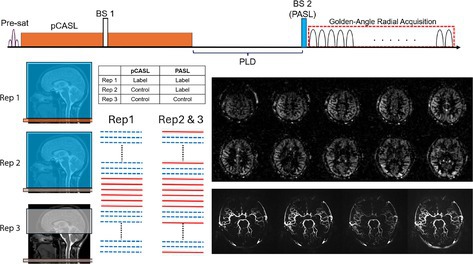

**Abbreviations**
4D MRA: four‐dimensional magnetic resonance angiography
ASL: arterial spin labeling
BS: background suppression
CBF: cerebral blood flow
ce‐MRA: contrast‐enhanced magnetic resonance angiography
DCE: dynamic contrast enhanced
DSC: dynamic susceptibility contrast
FAIR: flow‐sensitive alternating inversion recovery
MIP: maximum intensity projection
MRA: magnetic resonance angiography
NUFFT: non‐uniform fast Fourier transform
PASL: pulsed ASL
pCASL: pseudo‐continuous ASL
PLD: post‐labeling delay
SOS: stack of stars

## 1 Introduction

Comprehensive characterizations of cerebral hemodynamics in both macrovascular and microvascular systems are essential for the clinical diagnosis, treatment planning, and follow‐ups of cerebrovascular diseases [1, 2, 3, 4]. In conventional clinical practice, cerebral blood vessels and downstream perfusion are typically characterized by separate scans. For example, dynamic contrast‐enhanced MR angiography (ce‐MRA) is commonly employed to delineate flow dynamics in cerebral blood vessels, while dynamic susceptibility contrast (DSC) MRI and dynamic contrast‐enhanced (DCE) MRI are clinically used to detect perfusion deficits in cerebrovascular disease (e.g., stroke) and brain tumors [5, 6, 7]. These techniques require the administration of gadolinium contrast agents, which typically involve separate contrast injections, raising concerns about gadolinium contrast deposition in the brain [8, 9]. The administration of contrast also necessitates vascular access and takes extra time. Furthermore, inter‐scan head movement may complicate the diagnosis. Therefore, a single MRI acquisition without contrast injection that provides both angiographic and perfusion contrasts in the same spatial domain can streamline the clinical workflow by enhancing scan efficiency and reducing inter‐scan motion, and the intrinsically co‐registered MRA and perfusion images can enhance diagnostic confidence and efficiency, particularly in motion‐prone patients.

Arterial spin labeling (ASL) is a non‐invasive MRI technique that utilizes magnetically labeled arterial blood as an endogenous tracer [10, 11, 12] and has recently become favorable for cerebrovascular imaging, including non‐contrast enhanced MRA and perfusion imaging [13, 14, 15]. With pseudo‐continuous ASL (pCASL) and background suppression, ASL permits reliable cerebral blood flow (CBF) measurements with high labeling efficiency and signal‐to‐noise ratio (SNR), largely facilitating ASL perfusion imaging in clinical applications [16, 17, 18]. On the other side, with recent developments in both acquisition and image reconstruction, ASL‐based four‐dimensional MRA (4D MRA) has shown comparable performance to the conventional ce‐MRA techniques and has demonstrated effectiveness in examining cerebrovascular diseases [15, 19, 20, 21]. Although promising, current ASL techniques primarily focus either on angiography or on perfusion imaging. Separate sequences are typically required to make a comprehensive evaluation of brain vascular hemodynamics. Recent advancements in ASL have shown the feasibility of simultaneously acquiring angiographic and perfusion contrasts within a single scan, such as time‐encoded approaches and CAPRIA [22, 23, 24, 25], by employing a single pCASL labeling for both dynamic MRA and perfusion imaging.

In this work, we propose a dual‐module ASL technique to simultaneously obtain time‐resolved 4D angiography and quantitative perfusion images within a single scan. This technique integrated both pCASL and pulsed ASL (PASL) modules with a stack‐of‐stars (SOS) golden‐angle radial readout, by which the labeled spins from pCASL and PASL were decoded to generate perfusion and angiographic contrasts, respectively. To evaluate the technical feasibility, both numerical simulations and in vivo experiments were conducted. 4D MRA from the dual‐module ASL technique was systematically compared against a previously developed PASL‐based SOS golden‐angle radial 4D MRA method [26], and CBF quantifications from the perfusion images at different post‐labeling delays (PLD) were evaluated by comparison with a conventional pCASL sequence with background‐suppressed 3D GRASE readout.

## 2 Methods

### 2.1 Pulse Sequence Design

**FIGURE 1:**
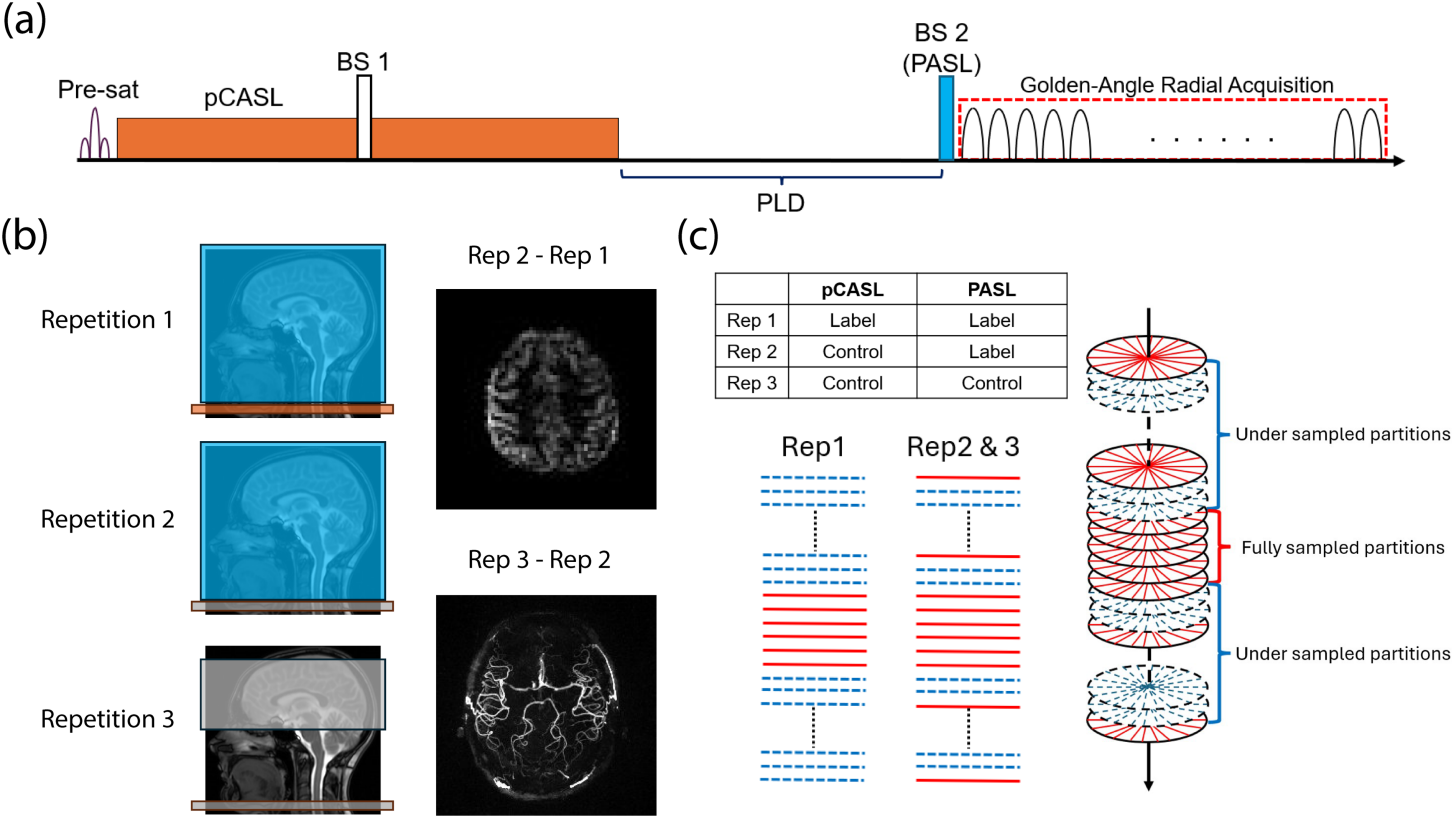
Pulse sequence and acquisition diagram of the proposed dual‐module ASL technique. (a) A background suppression (BS) module containing a pre‐saturation pulse and two inversion pulses is used to null longitudinal magnetization at the beginning of the sequence and suppress tissue signals. (b) The second inversion pulse served dual purposes for both background suppression and flow‐sensitive alternative inversion recovery (FAIR) PASL labeling, applied as either a slab‐selective or non‐selective pulse in each repetition. Label and Control conditions are altered in each repetition for pCASL and PASL preparations, where orange and blue boxes indicate Label conditions, and gray boxes indicate Control conditions for pCASL and PASL, respectively. Perfusion and 4D MRA are generated with paired subtractions. (c) Stack‐of‐stars golden‐angle radial bSSFP readout with partition direction undersampling is employed, where central partitions are fully sampled, and periphery partitions are undersampled with an acceleration factor of 3 (red solid line and blue dashed line indicate collected and skipped partitions, respectively). Only fully sampled central partitions are collected in the first repetition per partition for perfusion imaging.

The schematic of the proposed dual‐module ASL sequence is illustrated in Figure 1a. For magnetization preparation, a background suppression (BS) module was implemented, consisting of a slab‐selective pre‐saturation pulse at the beginning of each TR to null any residual signals, followed by two inversion pulses to suppress background tissue signals and their fluctuations. A pCASL labeling pulse train was applied after the pre‐saturation pulse to label inflowing arterial blood spins. The two inversion pulses were timed for background suppression, including the first inversion pulse applied within the pCASL pulse train and the second applied immediately before image acquisition to suppress background tissue signal to 10% at the midpoint of the readout train used for perfusion imaging. Note that the second inversion pulse served dual purposes: Except for BS, it acted as a flow‐sensitive alternating inversion recovery (FAIR) PASL labeling pulse [27], switching between non‐selective (blue region) and slab‐selective (gray region covering the brain) among repetitions, corresponding to PASL label and control states, respectively (Figure 1b).

An SOS golden‐angle (111.246°) radial [28] bSSFP readout was employed right after the second BS inversion pulse for image acquisition. Per partition encoding (TR), after magnetization preparation, a total of 380 in‐plane radial spokes were collected. Three interleaved repetitions were performed per partition, with label/control conditions of pCASL and PASL designed as shown in Figure 1b,c, before proceeding to the next partition. ASL signals from pCASL and PASL were disentangled from the encoded acquisitions: Subtraction between Repetitions 1 and 2 yielded cerebral perfusion, and subtraction between Repetitions 2 and 3 yielded MRA (Figure 1c).

To accelerate image acquisition, parallel imaging in the partition encoding direction was applied with fully sampled central partitions (red solid lines) and undersampled peripheral partitions (blue dashed lines) (Figure 1c). Note that the first repetition was used exclusively for perfusion; thus, only fully sampled central partitions were collected to further minimize total scan time. In contrast, peripheral partitions in Repetitions 2 and 3 were acquired to form high spatial resolution 4D MRA. Specifically, the sequence loops between Repetitions 2 and 3 for peripheral partitions, while in central partitions, the sequence loops through all three repetitions.

### 2.2 Numerical Simulations

Numerical simulations using the Bloch equations were performed in MATLAB (MathWorks, USA) to optimize the BS and evaluate ASL signal evolution along the pCASL module combined with the BS. A discrete time step of 1 ms and instantaneous RF excitation were assumed. For the BS, the dynamic tissue signal Mz(t) was simulated, considering longitudinal relaxation times of gray matter ($T_{1\mathrm{GM}}=1300\;\mathrm{ms})$, white matter $\left(T_{1\mathrm{WM}}=800\;\mathrm{ms}\right)$, and cerebrospinal fluid $\left(T_{1\mathrm{CSF}}=3600\;\mathrm{ms}\right)$. A pre‐saturation pulse nulling Mz was applied at the beginning, and the second inversion pulse was fixed right before readout at TI_2_ = 2500 ms. The time of placing the first inversion pulse was determined through the simulation of Mz(t) by having Mz (*t* = 2972 ms) of gray matter reach 0.1 Mz(0) at the acquisition of the central radial spoke (the 100th radial spoke) used for perfusion‐weighted image reconstruction. According to the simulation, the first inversion pulse was placed at TI_1_ = 1309 ms, which was within the pCASL pulse train. The inclusion of this inversion pulse separates the pCASL module into two segments, with the label/control condition of the latter segment flipped in all repetitions. The influence of readout was considered in tissue signal behaviors.

The longitudinal magnetization of ASL bolus ($\begin{array}{c} S_{\mathrm{ASL}}\left(t\right)=S_{\text{Control}}\left(t\right)-S_{\text{Label}}\left(t\right) \end{array}$) was simulated by taking the accumulation of blood spins over a labeling of 1800 ms, with a nominal PLD = 700 ms and assuming 5% signal loss from the background suppression pulses. An arterial transit time of 1.2 s was assumed [29]. The ASL signal attenuation during the readout train was also taken into account, as the blood signal S ($S_{\text{Control}},\;S_{\text{Label}}$) experienced apparent T_1_ relaxation, as shown in Equations ((1), (2)–3) [30, 31].

(1) $$S\left(t\right)=S_{\mathrm{ss}}-\left(S_{\mathrm{ss}}-S_{0}\right)\cdot e^{-\frac{t}{T_{1\mathrm{app}}}}$$

(2) $$T_{1\mathrm{app}}=\frac{1}{\left(\frac{\cos^{2}\frac{\theta }{2}}{\;T_{1b}}+\frac{\sin^{2}\frac{\theta }{2}}{T_{2b}}\right)}$$

(3) $$S_{\mathrm{ss}}=\frac{\cos\frac{\theta }{2}\cdot \sin\frac{\theta }{2}\cdot T_{1\mathrm{app}}}{\;T_{1b}}$$

where $T_{1\mathrm{app}}=1089\;\mathrm{ms},$ representing the apparent longitudinal relaxation time of blood calculated with $T_{1b}=1650\;\mathrm{ms}$ and $T_{2b}=190\;\mathrm{ms}$; $S_{0}$ is the initial blood signal right before the readout; $S_{\mathrm{ss}}$ is the steady‐state signal of blood achieved during the bSSFP readout RF pulse train; and $\theta =30^{{}^{\circ}}$ is the flip angle used for RF excitation. For the sake of simplicity, the transition phase at the beginning of the bSSFP readout train to achieve the steady state was not simulated.

### 2.3 MRI Experiments

Eleven healthy participants (7 males, 27.2 ± 3.82 years) were recruited in this study after obtaining local institutional review board approval and written informed consent. MRI scans were conducted on a Siemens 3T Prisma system (Siemens Healthcare, Erlangen, Germany) using a 64‐channel head coil.

A 3D time‐of‐flight (TOF) scan was performed as an anatomical reference for positioning the dual‐module ASL sequence. The imaging slab of the dual‐module ASL sequence covered the entire brain cortex and vessels above the Circle‐of‐Willis; the labeling plane was placed above the carotid artery bifurcation and below the V3 segment [32]. Three nominal PLDs (nPLDs), including 500, 700, and 1000 ms, were tested in 9 out of the 11 participants (6 males, 27.1 ± 4.08 years) with a total scan time of 6.43, 7.02, and 7.50 min, respectively, and only nPLD = 700 ms for the rest of the participants. A proton density (PD) scan (M_0_) was acquired separately using the same sequence but with only Repetition 1 and without any preparation modules. A deadtime of 3 s was added by the end of each TR to ensure adequate time for T1 recovery (scan time = 1.75 min). To evaluate 4D MRA performance, the proposed dual‐module ASL technique was compared with a previously developed 4D MRA sequence [26] in 7 out of the 11 participants (5 males, 25.7 ± 1.89 years). Slightly different from the previous one, the reference 4D MRA employed a FAIR‐based PASL labeling scheme applied immediately after the pre‐saturation pulse, followed by the same readout with imaging parameters matched to the proposed technique. To evaluate the feasibility of CBF quantification using the proposed technique, a conventional background‐suppressed pCASL perfusion sequence with segmented 3D GRASE readout was conducted in all participants as a reference with an EPI factor of 63, turbo factor of 14, and flip angle of refocusing pulses = 120° [17]. The imaging parameters of the three sequences are provided in Table 1. The CBF quantification was further evaluated on four additionally recruited participants (4 males, 25.5 ± 2.89 years) and compared against the pCASL 3D GRASE sequence with the same labeling duration of 1.8 s and slice thickness of 4 mm. The other imaging parameters of 3D GRASE followed the ASL consensus paper [17], including PLD = 1.8 s, in‐plane voxel size = 3.5 × 3.5 mm^2^, 24 slices with 15% oversampling, turbo factor = 8, EPI factor = 14, flip angle of refocusing pulses = 120°, TE/TR = 36.1/3990 ms, and eight pairs of label/control with a total scan time of 4 min 19 s.

**TABLE 1:** The imaging parameters of the proposed dual‐module ASL technique, the reference 4D MRA, and the 3D GRASE pCASL perfusion imaging.

Parameters	Dual‐module ASL	Reference 4D MRA	3D GRASE pCASL perfusion
Field of view (mm^3^)	224 × 224 × 84	224 × 224 × 84	224 × 224 × 84
Voxel size (mm^3^)	1 × 1 × 1.5	1 × 1 × 1.5	3.5 × 3.5 × 3
Slices	56	56	28
Slice oversampling (%)	14.3	14.3	14.3
TR (ms)	4496	1790	3800
TE (echo spacing) (ms)	2.34 (4.7)	2.34 (4.7)	36.44
Flip angle (°)	30	30	90
Bandwidth (Hz/pixel)	558	558	3004
Radial views	380	380	—
Undersampling factor	1.78	1.78	—
Fully sampled partitions	22	22	—
Label duration (ms)	1800	—	1500
Nominal post‐labeling delay (ms)	700	—	1600
Repetitions	1	1	12 (5 pairs + M0 scan)
Scan time (min)	7.02	3.25	2.28

### 2.4 Image Reconstruction

The raw k‐space data were transferred to an external server (Linux 7; CPU: Intel Xeon Gold 6338, 2 GHz; GPU: NVIDIA RTX A6000; RAM: 192 GB) for offline reconstruction using in‐house MATLAB programs. Two image sets, including 4D MRA and perfusion‐weighted images, were reconstructed independently.

#### 2.4.1 Reconstruction of 4D MRA

4D MRA images were reconstructed following these steps:

Step 1: For the multi‐channel raw k‐space data from the second and third repetitions, GRAPPA [33] was applied along the kz (partition encoding) direction to restore undersampled partitions. This process was performed independently for each radial angle. The postprocessed data were then compressed into eight virtual channels to reduce the total matrix size.
Step 2: A fast Fourier transform (FFT) was performed along the kz direction. The 380 radial spokes per slice were binned into 10 and 20 radial spokes per frame, yielding a temporal resolution of 47 and 94 ms/frame (in‐plane acceleration rate = 35.2 and 17.6), respectively. The data were then reconstructed using a previously developed self‐calibrated low‐rank subspace reconstruction method [26].
(4) $$\begin{array}{c} \tilde{V_{k}}={\text{argmin}}_{{\tilde{V}}_{k}}\frac{1}{2}{\left\|E\left(U_{k}V_{k}\right)-\sqrt{W}y\right\|}_{2}^{2}+\lambda_{1}{\left\|S_{s}V_{k}\right\|}_{1}+\lambda_{2}{\left\|S_{t}\left(U_{k}V_{k}\right)\right\|}_{1}\\ \;+\lambda_{3}{\left\|\left|U_{k}V_{\mathrm{kc}}\right|-\left|U_{k}V_{\mathrm{kl}}\right|\right\|}_{1} \end{array}$$
  where $U_{k}$ is the temporal basis (eigenvectors) estimated from the k‐space center; $V_{k}$ is the spatial basis decomposed from the whole k‐space data using the temporal basis, and $V_{\mathrm{kc}}\;\;\text{and}\;V_{\mathrm{kl}}$ are specifically for control and label datasets; $E=\sqrt{W}\mathrm{FC}$ represents the encoding operator for the multi‐coil k‐space interpolated FFT reconstruction; W represents the weighting parameters for k‐space interpolation from GROG [34], and F represents FFT; coil sensitivity maps C were generated with the adaptive array‐combination technique [35] from the fully sampled image combining all radial spokes using non‐uniform fast Fourier transform (NUFFT) reconstruction [35, 36]; y shows the k‐space data, which is in the cartesian trajectory interpolated by GROG; $S_{s}$ and $S_{t}$ are the spatial and temporal sparsifying transforms considered in the L1‐norm total variation constraint, and a magnitude difference constraint is applied between label and control images as represented by an L1‐norm total variation approach for denoising based on the similarity of adjacent frames, neighboring voxels, and label/control images; and $\lambda_{1},\lambda_{2},\text{and}\;\lambda_{3}$ are the regularization parameters for the spatial and temporal sparsity and magnitude difference controlling the tradeoff between data consistency and sparsity. $\lambda_{1}=0.001,\lambda_{2}=0.0008,\text{and}\;\lambda_{3}=0.00003$ were empirically selected for all subjects based on visual inspection of image quality and temporal fidelity in three test datasets. The cost function was minimized using the limited‐memory Broyden–Fletcher–Goldfarb–Shanno (L‐BFGS) algorithm, as in previous work [37, 38].
Step 3: Pair‐wise subtraction between the reconstructed label and control images yielded dynamic angiograms. Maximum intensity projection (MIP) was applied to the subtraction images for each frame along the transverse, coronal, and sagittal directions to visualize dynamic MRA.

For comparison, the raw k‐space data from the reference 4D MRA sequence were reconstructed using the same workflow and parameter settings as stated in Steps 1–3.

#### 2.4.2 Reconstruction of Perfusion‐Weighted Images

Perfusion‐weighted images were reconstructed as follows:

Step 1: K‐space data from the central 16 partitions within the fully sampled regions of the first and second repetitions were extracted for perfusion reconstruction, resulting in an effective slice thickness of 6 mm. These datasets were then preprocessed with the same coil compression method and kz direction FFT as used in 4D MRA reconstruction.
Step 2: The first 200 radial spokes from each partition and the central 64, 96, and 128 data points (out of a total 224) from each radial spoke were used for reconstruction, resulting in effective in‐plane resolutions of 3.5 × 3.5 mm^2^, 2.33 × 2.33 mm^2^, and 1.75 × 1.75 mm^2^, respectively. The extracted datasets were then processed using a compressed sensing reconstruction [37] with a spatial sparsity constraint applied through the total variation transform as follows:
(5) $$\tilde{m}=\text{argmin}\left\{{\left\|\text{NUFFT}\cdot C\cdot m-y\right\|}_{2}^{2}+\lambda {\left\|S_{s}m\right\|}_{1}\right\}$$
  where $\tilde{m}$ represents the label/control image series; C denotes the coil sensitivity maps generated using the same method as of 4D MRA; y is the collected k‐space data; $S_{s}$ represents the finite difference operator applied in the image domain; and λ is the regularization parameter for the spatial sparsity term. The $\lambda_{64}=0.0003,\lambda_{96}=0.004,\text{and}\;\lambda_{128}=0.03$ were empirically chosen for spatial resolution of 3.5 × 3.5 mm^2^, 2.33 × 2.33 mm^2^, and 1.75 × 1.75 mm^2^, respectively. The same optimizer was used as in the 4D MRA reconstruction.
Step 3: The pair‐wise subtraction between the reconstructed label and control images yields perfusion‐weighted images. For the M0 calibration, a series of PD‐weighted images was reconstructed, with 200 radial spokes per frame and a sliding window of 10 radial spokes. The same regularization weighting as used in Step 2 was applied.

The label and control images from the reference 3D GRASE pCASL were obtained directly from the vendor‐provided inline reconstruction. The perfusion‐weighted images were generated via control/label subtraction. Different from the proposed method, the perfusion‐weighted images from 3D GRASE pCASL were postprocessed with motion correction and 3D PCA denoising [39] prior to CBF quantification to improve the reliability of CBF measurement.

### 2.5 Image Evaluation

#### 2.5.1 4D MRA

To evaluate the feasibility of the dual‐module ASL technique in the depiction of arterial blood flow dynamics, structural similarity (SSIM) was used to compare the proposed technique with the reference 4D MRA. This metric evaluates the consistency in blood contrast and vessel delineation, and a higher SSIM indicates better similarity in arterial blood flow dynamics, image contrast, and overall image quality, which was calculated by the following equation:

(6) $$\text{SSIM}\left(x,y\right)=\frac{\left(2\mu_{x}\mu_{y}+c_{1}\right)\left(2\sigma_{\mathrm{xy}}+c_{2}\right)}{\left(\mu_{x}^{2}+\mu_{y}^{2}+c_{1}\right)\left(\sigma_{x}^{2}+\sigma_{y}^{2}+c_{2}\right)}$$

where $\mu_{x}$, $\mu_{y}$ $\sigma_{x}^{2}$, and $\sigma_{y}^{2}$ $\sigma_{\mathrm{xy}}$ are the mean, variance, and covariance of pixel intensities of image *x* and *y*; $c_{1}\;\text{and}\;\;c_{2}$ are the constants added to prevent zero denominators. SSIM was calculated on MIP images along transverse, coronal, and sagittal views per frame using the reconstruction with 20 radial spokes per frame. Before the calculations, the intensities of MIP images were first normalized across entire frames, so that both techniques shared the same intensity range, and a brain mask was applied to exclude the background region outside the brain, which was generated via an intensity thresholding on the original control images from the proposed technique. The final SSIM was averaged across all frames and subjects.

To better compare image quality, SNR maps were generated from collapsed MIP (cMIP) images across all frames for both the proposed and the reference 4D MRA. A 20 × 20 pixel region in the upper‐left corner of the image was chosen as the background for SNR calculation.

In addition, blood dynamic curves were extracted from the 4D MRA images. Four regions of interest (ROI) were manually drawn on the M1 and M2/3 segments of the middle cerebral artery (MCA) and the P1 and P2 segments of the posterior cerebral artery (PCA). The correlation coefficients of blood dynamics curves between the proposed technique and the reference were calculated and averaged across all subjects, and averaged dynamic curves for each ROI were generated for visualization. A higher correlation coefficient indicates good similarity in arterial blood flow dynamics.

#### 2.5.2 CBF Quantification

CBF quantification was performed using a single‐compartment model [40] as follows:

(7) $$\mathrm{CBF}=\frac{6000\cdot \lambda \cdot ∆S\cdot e^{\frac{\mathrm{PLD}}{T_{1b}}}}{2\cdot \alpha \cdot T_{1b}\cdot S_{M0}\cdot \left(1-e^{-\frac{\tau }{T_{1b}}}\right)}$$

where the λ=0.9mL/g is the blood/tissue water partition coefficient; $∆S=S_{\text{Control}}-S_{\text{Label}}$ represents the perfusion‐weighted signal. For the proposed technique, the effective PLD was calculated as follows: effective PLD = nPLD + effective readout duration, defined as the time to reach the averaged signal attenuation (e.g., 472 ms in reconstruction using 200 radial spokes); three nPLD settings, including 500, 700, and 1000 ms, were evaluated (effective PLD of 972, 1172, and 1472 ms). $T_{1b}=1650\;\mathrm{ms}$ is the longitudinal relaxation value of blood; α=0.767 is the labeling efficiency after considering two background suppression pulses; $S_{M0}$ represents the equilibrium magnetization. For each brain voxel, it is estimated by fitting the series of reconstructed PD weighted images to an exponential function; τ is the pCASL labeling duration.

Similar to previous work [41], to account for signal attenuation caused by the series of RF pulses during the bSSFP readout train, the final CBF was corrected for the average attenuation effect across all 200 radial spokes, using an attenuation coefficient as follows:

(8) $${\mathrm{CBF}}_{\text{corrected}}=\frac{\mathrm{CBF}}{c}$$

(9) $$c=\sum_{i}^{N}\frac{{\left(e^{-\frac{\mathrm{TR}}{T_{1b}}}{\cdot \cos}^{2}\frac{\theta }{2}+e^{-\frac{\mathrm{TR}}{T_{2b}}}\cdot \sin^{2}\frac{\theta }{2}\right)}^{i}}{N}$$

where $\theta =30^{{}^{\circ}}$ is the flip angle of RF excitation in the bSSFP readout; N=200 is the number of radial spokes for perfusion reconstruction; and $T_{1b}=1650\;\mathrm{ms}$ and $T_{2b}=190\;\mathrm{ms}$ are the relaxation values of arterial blood.

The average CBF values in gray matter were calculated from both the proposed technique and the reference for all subjects. The gray matter masks were obtained from the M0 images using segmentation on SPM12 (UCL, UK) with a probability threshold of > 0.7, individually. A one‐way repeated measures analysis of variance (ANOVA), followed by paired *t*‐tests, was performed to evaluate any group difference in mean CBF values between the proposed technique and the reference and among different PLD settings. A two‐tailed *p*‐value of less than 0.05 indicated statistical significance.

## 3 Results

**FIGURE 2:**
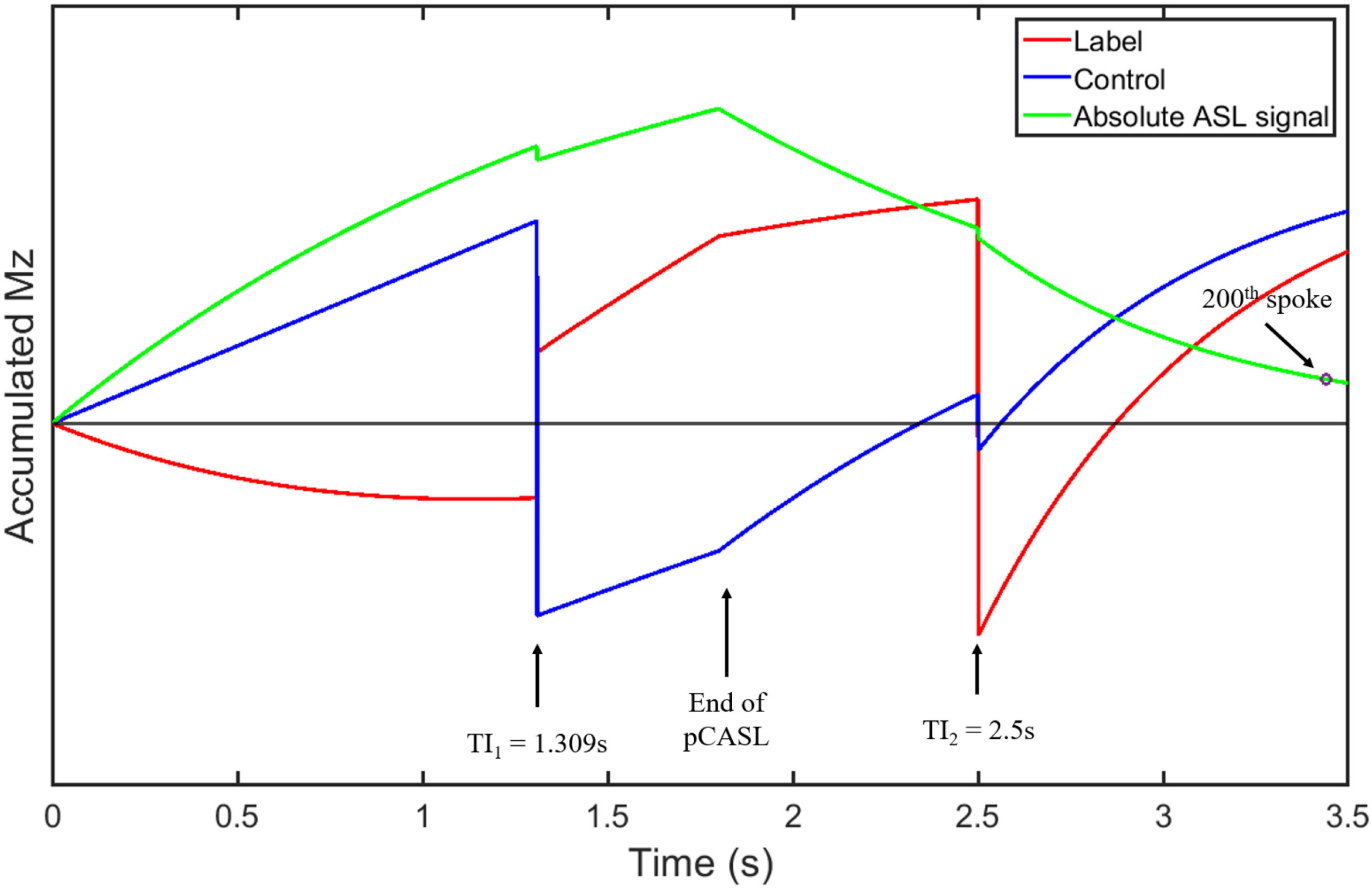
Numerical simulation of blood signal behavior from the pCASL module under Label and Control conditions, as well as the absolute ASL bolus. The first inversion pulse in the background suppression module was applied at TI_1_ = 1309 ms, dividing the pCASL module into two segments with flipped label and control conditions. The second inversion pulse was set immediately before the readout at TI_2_ = 2500 ms. At the time of acquiring the 200th radial spoke, ~16% of the ASL bolus signal remained.

The simulated signal evolutions of pCASL bolus, as well as control and label conditions, along the magnetization preparation and image acquisition, are shown in Figure 2. The ASL signal behavior followed the general kinetic model. With an nPLD = 700 ms, approximately 16% of pCASL signals remained at the 200th radial spoke during acquisition, which was used as the cutoff for selecting the number of radial spokes used for perfusion reconstruction. The tissue signal behaviors under the BS are shown in Figure S1.

**FIGURE 3:**
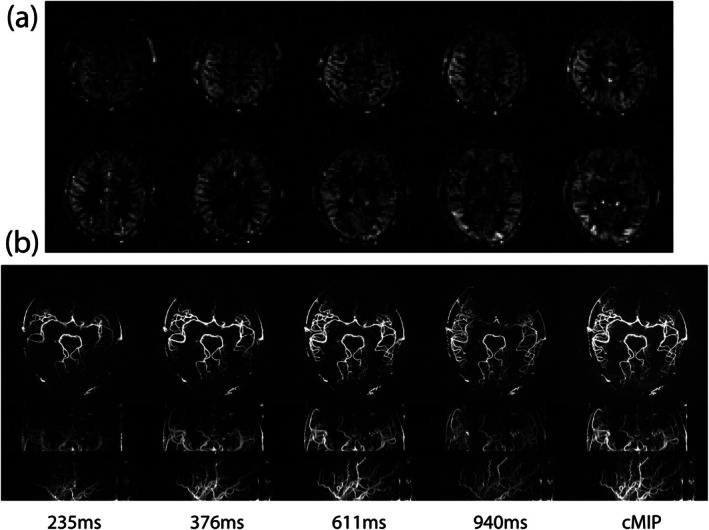
Reconstructed images from the proposed technique in a representative case with nominal PLD of 700 ms. (a) Perfusion‐weighted images across several slices, showing good perfusion contrast with whole brain coverage. (b) 4D MRA MIP images from several selected temporal frames and cMIP images in transverse, sagittal, and coronal views reconstructed using 10 radial spokes per frame. The proposed technique enables the visualization of complete blood flow dynamics through the cerebral vasculature.

Figure 3 shows the reconstructed images from the proposed technique in a representative case with nPLD = 700 ms. Figure 3a displays the reconstructed perfusion‐weighted images across several slices. Overall, good perfusion contrast was appreciated throughout the brain cortex. Figure 3b shows the selected 4D MRA MIPs and cMIP across phases reconstructed using 10 radial spokes per frame (47 ms/frame) in transverse, sagittal, and coronal views, revealing the full passage of dynamic blood flow, including both inflow and outflow phases with high spatiotemporal resolution and good image quality. The entire passage of dynamic labeled blood flow can be appreciated in Video S1. These results demonstrate the feasibility of the proposed technique for capturing both perfusion and dynamic angiography within a single scan.

**FIGURE 4:**
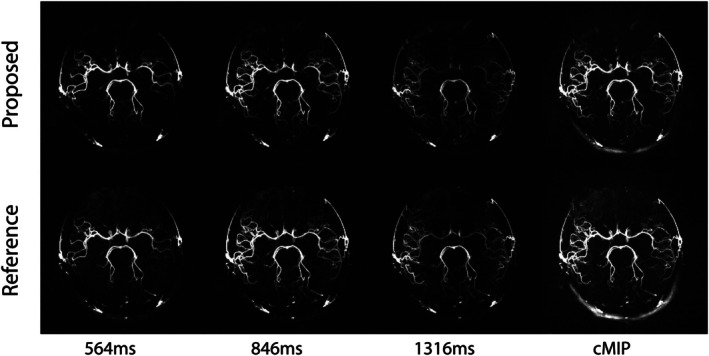
Several representative frames of MIP and cMIP images from the proposed technique and the reference 4D MRA reconstructed using 20 radial spokes per frame. Nearly identical flow dynamics and comparable image quality can be appreciated between the two techniques, supporting the feasibility of the proposed technique for 4D MRA and demonstrating the absence of interference between the two ASL modules used in the sequence.

**FIGURE 5:**
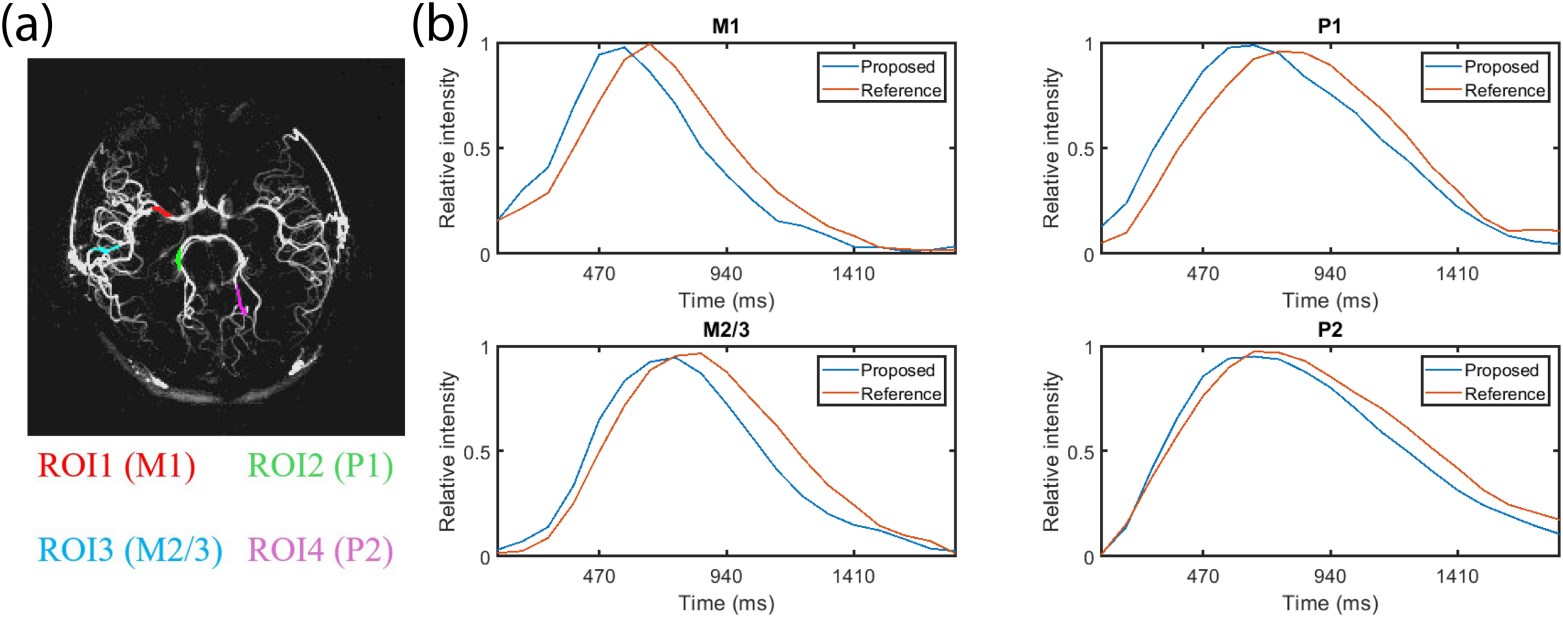
(a) Example of manually selected ROIs in M1 and M2/3 segments of MCA, and P1 and P2 segments of PCA. (b) Subject‐level averaged dynamic signal curves in four ROIs from the proposed technique and the reference 4D MRA. Similar dynamic curves are achieved across all ROIs, with a slight temporal shift, particularly in major branches, which might relate to the complication of the temporal basis estimated in subspace reconstruction.

Figure 4 compares 4D MRA MIP and cMIP images reconstructed with 20 radial spokes per frame (94 ms/frame) using the dual‐module ASL technique and the reference 4D MRA. The 4D MRA from the proposed technique exhibits dynamic blood flow patterns nearly identical to the conventional method, indicating that the ASL signals contributing to angiographic contrast are solely from the PASL module without interference from the pCASL modules. Additionally, the 4D MRA images from the proposed technique show comparable or even improved image quality, with a relatively clear background, likely to benefit from background suppression. These visual inspections were further confirmed by the quantitative evaluation (Table 2), with an average SSIM of 0.95 in the transverse MIP images and an SSIM of 0.9 in the coronal and sagittal MIP images. Figure 5 illustrates four ROIs from MCA and PCA on the cMIP image (Figure 5a) and the corresponding subject‐level averaged dynamic MRA signal curves from the proposed technique and reference (Figure 5b). Good similarity in blood dynamics was achieved between the two methods with high correlation coefficients of 0.933, 0.950, 0.918, and 0.977 for M1, M2/3, P1, and P2, respectively (Table 2), although a marginal temporal shift was observed.

**TABLE 2:** Quantitative evaluation results of 4D MRA images.

SSIM	Transverse	0.951 ± 0.023
	Coronal	0.888 ± 0.033
	Sagittal	0.888 ± 0.035
Correlation coefficient	M1	0.933 ± 0.041
	P1	0.918 ± 0.055
	M2/3	0.950 ± 0.057
	P2	0.977 ± 0.020

**FIGURE 6:**
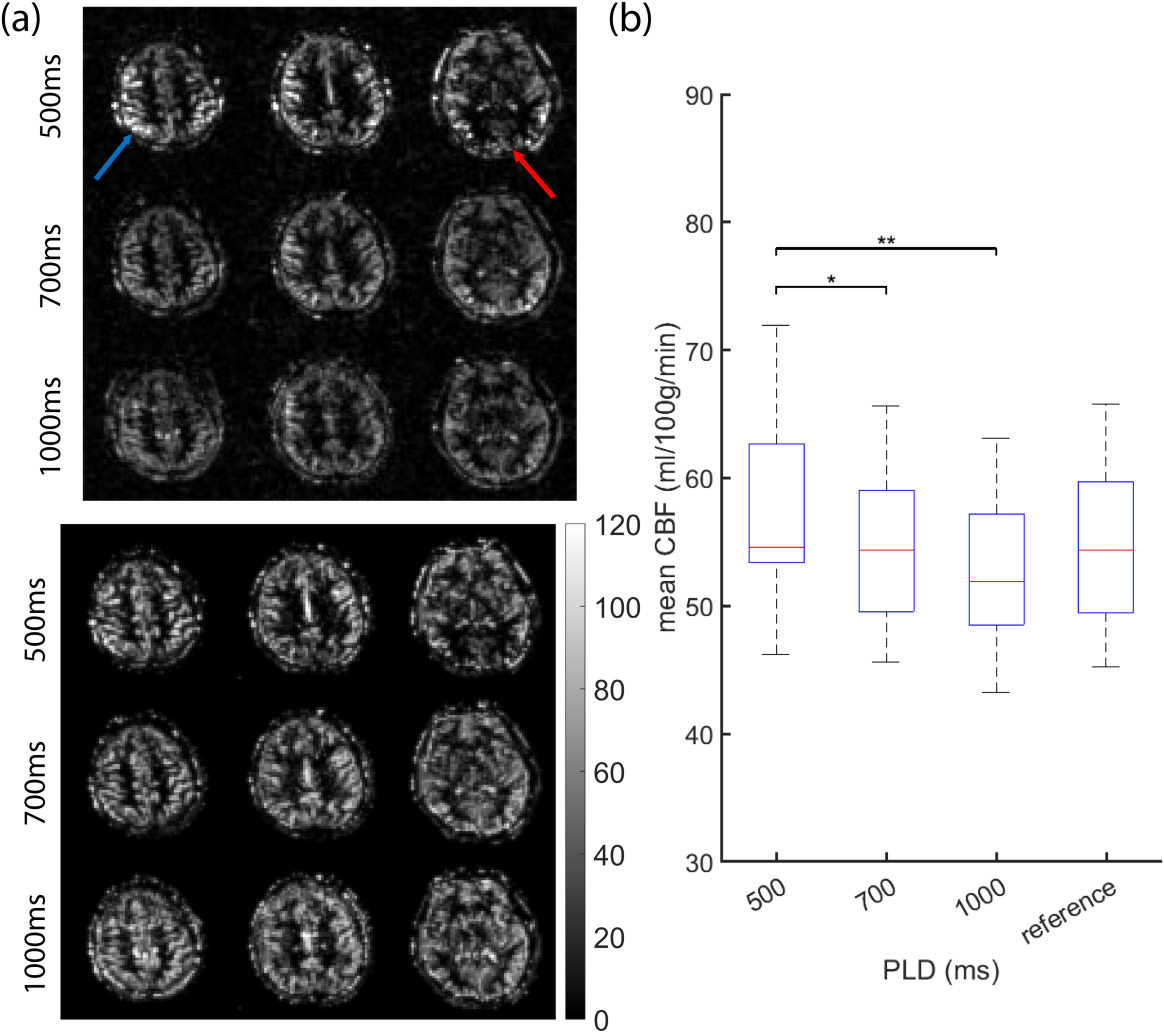
(a) Perfusion‐weighted images and the corresponding CBF maps from several slices with three different nominal PLD settings (500, 700, and 1000 ms). (b) Box plots of mean gray matter CBF values across nPLDs and reference (unit: mL/100 g/min). Some hyper‐ (blue arrow) and hypoperfusion (red arrow) regions can be found with the shorter nPLD of 500 ms, which shows statistically higher CBF values compared to two other nPLD settings (*p* = 0.021 and *p* = 0.008) but not with reference (*p* = 0.059). The CBF maps are more comparable between nPLDs of 700 and 1000 ms, and nPLD = 700 ms shows the closest CBF values as compared to the reference. The longer nPLD of 1000 ms shows slightly lower CBF values compared to the reference, although the difference is not statistically significant (*p* = 0.12).

Figure 6a shows the perfusion‐weighted images and corresponding CBF maps with three nPLD settings. Strong perfusion contrast between gray and white matter is evident for all PLDs. However, some arterial transit artifacts were observed with a shorter nPLD = 500 ms, appearing as bright serpiginous signals on perfusion maps, as indicated by the blue arrows. Meanwhile, some lower tissue perfusion was observed in the posterior brain regions, as indicated by the red arrows. In contrast, CBF maps exhibit more uniform gray matter distributions with reduced arterial transit artifacts with two longer nPLDs. Compared to those with nPLD = 700 ms, the perfusion‐weighted images with nPLD = 1000 ms present increased noise, likely suffering from stronger ASL signal decay. Figure 6b shows the box plots of the whole brain mean gray matter CBF values across subjects, with 57.93 ± 8.42, 54.44 ± 6.63, and 52.58 ± 6.61 mL/100 g/min for nPLDs of 500, 700, and 1000 ms, respectively. The mean CBF from 3D GRASE pCASL (CBF_ref_ = 54.74 ± 6.74 mL/100 g/min) was also obtained across subjects as a reference. Consistent with the visual inspection in Figure 6, the mean CBF values were greater with a shorter nPLD = 500 ms due to the contamination of macrovascular signals and significantly different from the CBF values with the other two nPLD settings (*p* = 0.021, 0.008), although there was no significant difference with the 3D GRASE reference (*p* = 0.059). The CBF values measured with the longer nPLD = 1000 ms tended to be smaller, despite no significant difference from the reference (*p* = 0.12). In contrast, CBF values with nPLD = 700 ms were most comparable to the reference, and total scan time was shorter than that with nPLD = 1000 ms. Therefore, nPLD = 700 ms was selected as the optimal setting in the dual‐module ASL in this work. Additionally, the box plots of comparison in mean CBF values between the proposed technique and the 3D GRASE reference using the recommended parameters are shown in Figure S2. No statistical differences were found between the two results (*p* = 0.10, paired *t*‐test).

**FIGURE 7:**
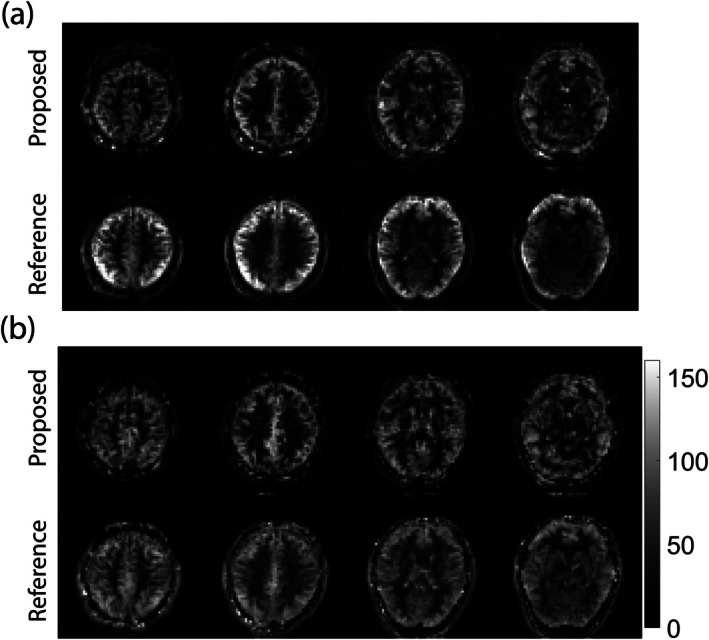
Comparisons of (a) perfusion‐weighted images and (b) corresponding CBF maps obtained from the proposed technique and the 3D GRASE pCASL reference (unit: mL/100 g/min). Perfusion‐weighted images from 3D GRASE were postprocessed after motion correction and 3D PCA denoising, while the ones from the proposed technique were directly generated from reconstructed images without additional postprocessing. Distinct perfusion contrasts are acquired, while comparable CBF quantifications are achieved between the two techniques in gray matter.

Figure 7 shows the comparisons of perfusion‐weighted images and corresponding CBF maps between the proposed technique with nPLD = 700 ms and the reference. Note that the displayed perfusion‐weighted images from 3D GRASE were postprocessed after motion correction and 3D PCA denoising [39], while the ones from the proposed technique were directly generated from reconstructed images without additional postprocessing. Similar CBF maps were obtained, although with slightly different perfusion contrast, especially in white matter. The corresponding comparison of CBF maps from the additionally collected data with the same labeling duration of 1.8 s is shown in Figure S3. Consistently, similar CBF measurements in gray matter can be observed, although the perfusion contrast between gray and white matter looks stronger with the proposed method.

**FIGURE 8:**
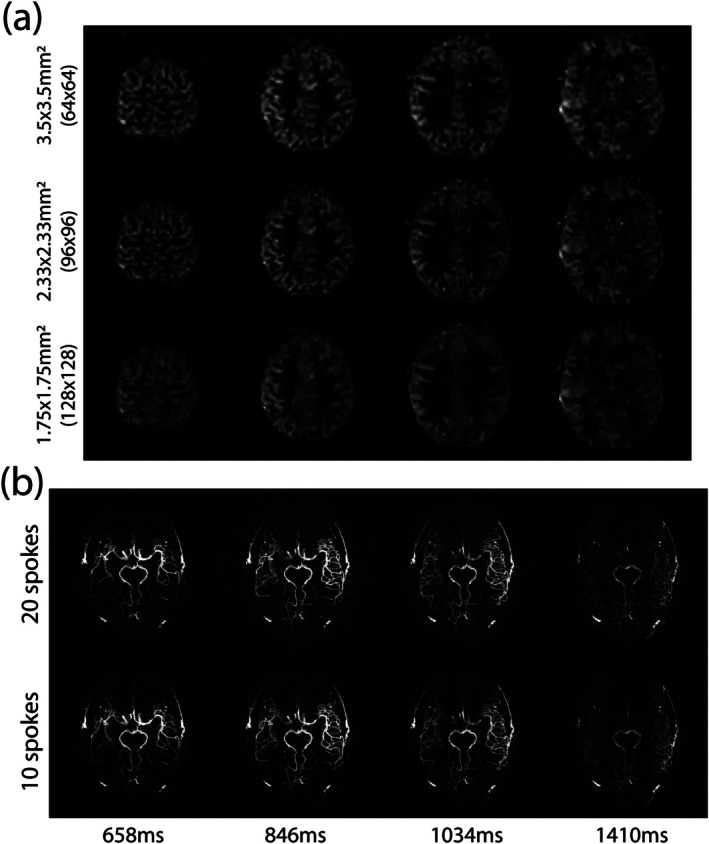
(a) Several representative slices of perfusion‐weighted images reconstructed at different effective spatial resolutions from the proposed technique. Higher regularization weightings were used for higher spatial resolution. Good perfusion contrasts are maintained across all spatial resolutions. (b) Several representative frames of 4D MRA reconstructed at different temporal resolutions from the proposed technique. Identical blood flow dynamics are observed without noticeable image degradation.

In the dual‐module ASL, radial acquisition enables flexible reconstruction of 4D MRA at different acceleration rates (temporal resolution) and perfusion images at varied spatial resolutions. Figure 8a illustrates the perfusion‐weighted images with three effective spatial resolutions displayed at the same window level. Good perfusion contrast was consistently observed across different resolutions. Higher spatial resolutions provided improved tissue boundary definition in perfusion maps with reduced partial volume effects, while higher regularization weighting was needed in order to achieve good image quality, which may in turn cause image blurring. Figure 8b presents the MIP images at several representative frames of the reconstructed 4D MRA using 20 and 10 radial spokes per frame. Clear visualization of both vascular structures and blood flow dynamics was obtained at both high acceleration rates without noticeable degradation in image quality.

## 4 Discussion

In this work, we introduced a novel ASL technique that combines dual‐module ASL preparation with SOS golden‐angle radial acquisition, enabling simultaneous acquisitions of dynamic 4D MRA and downstream cerebral perfusion from a single scan. The feasibility of the proposed technique in providing concurrent perfusion and MRA contrasts was demonstrated through both qualitative and quantitative comparisons with the reference techniques, showing comparable performance. These findings indicate the proposed dual‐module ASL technique a promising imaging technique, which provides high‐quality, high‐resolution time‐resolved angiography and quantitative perfusion maps within a single scan.

Recent efforts have been made to simultaneously acquire brain angiograms and perfusion maps using ASL. Suzuki Y et al. proposed a pCASL labeling scheme combined with time‐encoded technique [25], where 4D MRA at different temporal frames was generated through Hadamard decoding from multiple repetitions, and perfusion images were acquired from a separate readout train by the end of each repetition. However, in that approach, the temporal resolution of 4D MRA was predetermined, and perfusion signals potentially suffered from saturation effects caused by the prior 4D MRA readout. Another approach, CAPRIA [22, 23], combined pCASL with golden‐angle radial readout, enhancing the flexibility of temporal resolutions for dynamic MRA and perfusion imaging. Both MRA and perfusion contrasts were derived from the same preparation and readout by reconstructing different k‐space subsets. While it provides simplicity in sequence and flexibility in generating images with desired temporal information, the dynamic MRA and perfusion contrasts are potentially suffering from ASL signal attenuation because of the relatively long spoiled gradient echo readout train. By carefully designing the readout pulse train and modeling ASL signals, such an effect can be largely corrected and lead to robust CBF quantification [42]. Van der Plas et al. [43] proposed integrating time‐encoded pCASL with a sophisticated‐designed golden‐angle radial acquisition to alleviate these limitations but at a cost of limited slice coverage or resolution. Our technique is different from these methods, which incorporates pCASL with PASL labeling modules through modifying the spatial coverage of the second inversion pulse in the background suppression. Through decoding, the pCASL module with its integrated background suppression and PLD can effectively suppress background tissue signals and alleviate pCASL signal attenuation over the radial readout train, respectively, to provide robust perfusion imaging. Angiographic contrast can be obtained solely from the PASL module, which provides a complete inflow–outflow bolus passage compared to pCASL‐based dynamic MRA.

With the dual‐module ASL, high‐quality 4D MRA from the PASL module was obtained at different acceleration rates using the low‐rank subspace reconstruction. The delineations of arterial flow dynamics and vascular anatomy were consistent with the reference 4D MRA, as evidenced by high SSIM and correlation coefficient values. Figure S4 further confirmed this, as similar SNR maps were achieved, and most vascular regions maintained high SSIM on the cMIP image. A slight temporal shift of blood dynamics between the proposed technique and the reference was observed. This could be related to the different tissue behavior presented, where a combined tissue and blood temporal basis was used for subspace modeling in the reconstruction. This effect could bias blood dynamics, especially in major arteries with early inflow peaks. Further investigation will be needed to address this issue. CBF maps derived from the dual‐module ASL showed good perfusion contrast without notable arterial contamination after the nPLD optimization, in which the perfusion contrast was solely generated from the pCASL bolus, and the pCASL signal attenuation and mixed perfusion contrast along the radial readout were appropriately corrected. Similar CBF values were obtained compared to those from the 3D GRASE pCASL, suggesting that the dual‐module ASL technique allows for reasonable CBF quantification. Together, these findings demonstrate the feasibility of the proposed technique to provide high‐quality 4D MRA and quantitative perfusion imaging in a single scan without interference between PASL and pCASL labeling modules. Additionally, we compared SNR and SNR efficiency between the proposed technique and reference techniques (Table S1), where no statistical difference was found (*p* = 0.49 and *p* = 0.68 for perfusion images and *p* = 0.15 and *p* = 0.92 for MRA, for SNR and SNR efficiency, respectively; paired *t*‐tests). Note that the SNR from 3D GRASE perfusion was calculated from perfusion‐weighted images directly obtained from pair‐wise subtraction prior to any postprocessing, for a fair comparison with the proposed method. The SNR findings were further confirmed through the comparison between the proposed method and pCASL 3D GRASE with recommended parameters at the same spatial resolution (SNR: 20.28 ± 2.04 vs. 19.47 ± 0.43, *p* = 0.44; SNR efficiency: 0.99 ± 0.10 vs. 0.91 ± 0.02, *p* = 0.19; paired *t*‐tests, *n* = 4). However, the interpretation of SNR and SNR efficiency comparisons between the proposed method and pCASL with 3D GRASE should be made cautiously. When iterative reconstruction methods are used, noise characteristics depend on regularization parameters and reconstruction constraints. In addition, the readout is intrinsically distinct, leading to certain differences in the parameter settings.

There were several technical considerations in the design of the dual‐module ASL technique. Firstly, to isolate labeled spins between the two ASL modules, the label and control conditions of the pCASL and PASL modules were encoded among repetitions. However, instead of using a full Hadamard encoding matrix, only three repetitions were used in dual‐module ASL to reduce the total scan time. Alternatively, a full 4 × 2 Hadamard encoding strategy could be implemented to decode both MRA and perfusion, potentially boosting SNR and SNR efficiency, but at the expense of a 1.53‐fold longer scan time (144 vs. 94 partition encodings), which could reduce its clinical feasibility. Secondly, two strategies were taken to further reduce the total scan time. Parallel imaging was implemented along the partition direction, where GRAPPA [33] and a low‐rank subspace reconstruction [26] enabled reliable 4D MRA images with a high acceleration rate without compromising image quality. On the other hand, only central partitions in the first repetition were collected to generate perfusion contrast, leveraging the relatively intrinsic low spatial resolution of perfusion. Yet, the slice thickness of perfusion‐weighted images could potentially be improved, similar to the 4D MRA reconstruction, by acquiring peripheral partitions and applying the GRAPPA technique. Thirdly, given the fact that ASL signals dramatically attenuate when going into the microvasculature during the readout train, the first 200 radial spokes were used for perfusion reconstruction to guarantee a reliable CBF quantification. Considering that the arterial transit time (ATT) in healthy adults is around 1200 ms [29] and signal attenuates with repeated RF excitations, three nPLDs of 500, 700, and 1000 ms were tested with 200 radial spokes used in perfusion image reconstruction. Our results showed that, for an nPLD of 500 ms, part of the labeled bolus remained in the macrovascular space, causing arterial transit artifacts (Figure 6). With both longer nPLD, CBF maps became more uniform and comparable to the reference. nPLD of 700 ms was selected as the optimal nPLD, as it provided the best balance between image quality and CBF quantification while maintaining a reasonable acquisition time.

Different from conventional Cartesian acquisition used in ASL, radial acquisition inherently samples the k‐space center at each spoke, leading to a mixture of perfusion signals over an extended range of PLDs across the 200 radial spokes used for perfusion image reconstruction. For example, the PLD ranged from 700 to 1640 ms with an nPLD of 700 ms. To take this into account in the CBF quantification, an effective PLD was estimated by having an equal contribution of each radial spoke to the reconstructed image and applying a correction for signal decay. The three nPLDs of 500, 700, and 1000 ms with 200 radial spokes for perfusion readout resulted in effective PLDs of 972, 1172, and 1472 ms, respectively. Using effective PLDs, reasonable CBF quantification comparable to that of the reference was achieved with nPLDs ≥ 700 ms. Moreover, reconstructing perfusion images with fewer radial spokes centered around the desired PLD could also potentially mitigate such an effect (Figure S5), although no obvious difference was observed based on the results. Likewise, grouping radial spokes into temporal bins allows reconstruction of perfusion‐weighted images at multiple effective PLDs, analogous to CAPRIA [22, 23], which may also help correct such bias and potentially enable ATT quantification (Figure S6).

The proposed technique has limitations. Firstly, although bSSFP readout offers intrinsic high T2/T1 contrast and has lower ASL signal attenuation compared to spoiled gradient echo [44], it is sensitive to B0 field inhomogeneity, which was evident as signal loss in the ACA branches in some 4D MRA cases near tissue–air interfaces, similar to previous studies [15, 44, 45]. This could also cause signal loss in the perfusion‐weighted images and therefore affect CBF quantification, apparent as the banding artifact. Secondly, only gray matter CBF was evaluated through comparison with 3D GRASE pCASL. White matter perfusion was overall weaker with the proposed technique compared to the reference. This might be attributed to stronger ASL signal attenuations in the white matter with look‐locker readout. Thirdly, in the current study, the PLD was optimized for healthy young adults, which may not be optimal in different age groups or patient populations, where a longer nPLD may be needed for CBF quantification. Further optimization of the sequence design and imaging parameters is needed to enhance the reliability of CBF measurements at longer nPLDs in clinical applications. Additionally, technique validation in broad patient cohorts with different cerebrovascular diseases is necessary to establish its clinical utility. Fourthly, the slice thickness of the 3D GRASE reference was half that of the proposed technique. For visualization, down‐sampled 3D GRASE images in the slice dimension were generated, which may introduce some bias in the qualitative comparisons of perfusion‐weighted images, although the CBF quantification should not be much influenced by such a difference in spatial resolution. However, the additional comparison between the proposed technique and pCASL 3D GRASE with the same spatial resolution showed consistent results, demonstrating the robustness of CBF quantification using the proposed technique. Finally, as a proof‐of‐concept study, the current technique had a scan time approaching the combination of the two separate 4D MRA and pCASL scans, and it required off‐line reconstruction. Further optimization of the acquisition scheme and imaging parameters, as well as incorporating inline reconstruction framework, is needed in future work to facilitate its clinical utility.

In conclusion, this work introduced a dual‐module ASL technique for simultaneously acquiring 4D MRA and perfusion images within a single sequence. Specifically, a dual‐module ASL scheme including both pCASL and PASL is integrated with an SOS golden‐angle radial bSSFP readout. The proposed technique demonstrates the feasibility of acquiring high‐spatiotemporal resolution, time‐resolved 4D MRA, and quantitative perfusion images with flexible spatial resolution. This technique could be a useful imaging tool for comprehensively characterizing cerebral hemodynamics in both macrovascular and microvascular systems within a single scan.

## Author Contributions

Conceptualization, methodology, and experimental design: Zhao T. and Yan L. Data collection: Zhao T., Tang J., Moum S.J., He Y., Huang Z., Khan A., and Yan L. Image reconstruction: Zhao T. and Huang Z. Data analysis: Zhao T., Tang J., and He Y. Result interpretation: Zhao T., Tang J., Moum S.J., Shaibani A., and Yan L. Manuscript writing: Zhao T., Tang J., He Y., Huang Z., and Yan L. Manuscript revision: Zhao T., Moum S.J., Khan A., Shaibani A., and Yan L.

## Funding

This work was supported by the National Institute of Health (NIH) (grants R01NS118019, R01AG072490, and RF1NS139370) and BrightFocus Foundation (A20201411S).

## Conflicts of Interest

The authors declare no conflicts of interest.

## Supporting information


**Table S1:** Subject‐level averaged SNR and SNR efficiency results.
**Figure S1:**. Longitudinal magnetization evolution of tissue (gray matter, white matter, and cerebrospinal fluid) in the proposed technique, with the background suppression pulses applied at TI1 = 1309 ms, TI2 = 2500 ms, and considering the effect of readout.
**Figure S2:** Additional comparison of CBF between the proposed technique and the 3D GRASE reference using recommended parameters. No statistical difference was found in mean CBF values (*p* = 0.10, paired *t*‐test, *n* = 4).
**Figure S3:** Additional comparison of CBF maps between the proposed technique and the 3D GRASE reference using recommended parameters (unit: mL/100 g/min). Some signal loss in the frontal region from the proposed technique was observed because of banding artifact from bSSFP readout.
**Figure S4:** (a) Representative SNR maps of transverse cMIP images from the proposed technique and the 4D MRA reference. (b) Corresponding SSIM map comparing transverse cMIP images from the proposed technique and the reference, with vascular regions highlighted. Similar SNR maps were achieved, and most vascular regions maintained high SSIM on the cMIP image.
**Figure S5:** CBF maps reconstructed using 100 and 200 radial spokes with the same effective PLD, respectively. The same quantification methodology was implemented for perfusion results with 100 radial spokes. Similar quality and CBF values were obtained when comparing the reconstruction with different radial spokes.
**Figure S6:** Dynamic perfusion‐weighted images with different effective PLDs from three representative slices reconstructed with 60 radial spokes per frame. Local brightness was present in some distal vessels in the early phase, while the perfusion‐weighted images became more uniform in the later phase.

**Video S1:** Dynamic blood flow visualized as 4D MRA MIP images in three views.

## Data Availability

The data that support the findings of this study are available from the corresponding author upon request. The data are not publicly available because of privacy or ethical restrictions.
