# Supplementary material for: Concurrent Four‐Dimensional Dynamic MRA and Perfusion Imaging Using Dual‐Module Arterial Spin Labeling MRI With Stack‐Of‐Stars Golden‐Angle Radial Acquisition
Source: NMR Biomed. 2026 Jul 22;39(9):e70359. doi: 10.1002/nbm.70359 (PMC13392063; doi:10.1002/nbm.70359)
Supplement: Supplementary file 1 — Table S1: Subject‐level averaged SNR and SNR efficiency results. Figure S1:. Longitudinal magnetization evolution of tissue (gray matter, white matter, and cerebrospinal fluid) in the proposed technique, with the background suppression pulses applied at TI1 = 1309 ms, TI2 = 2500 ms, and considering the effect of readout. Figure S2: Additional comparison of CBF between the proposed technique and the 3D GRASE reference using recommended parameters. No statistical difference was found in mean CBF values (p = 0.10, paired t‐test, n = 4). Figure S3: Additional comparison of CBF maps between the proposed technique and the 3D GRASE reference using recommended parameters (unit: mL/100 g/min). Some signal loss in the frontal region from the proposed technique was observed because of banding artifact from bSSFP readout. Figure S4: (a) Representative SNR maps of transverse cMIP images from the proposed technique and the 4D MRA reference. (b) Corresponding SSIM map comparing transverse cMIP images from the proposed technique and the reference, with vascular regions highlighted. Similar SNR maps were achieved, and most vascular regions maintained high SSIM on the cMIP image. Figure S5: CBF maps reconstructed using 100 and 200 radial spokes with the same effective PLD, respectively. The same quantification methodology was implemented for perfusion results with 100 radial spokes. Similar quality and CBF values were obtained when comparing the reconstruction with different radial spokes. Figure S6: Dynamic perfusion‐weighted images with different effective PLDs from three representative slices reconstructed with 60 radial spokes per frame. Local brightness was present in some distal vessels in the early phase, while the perfusion‐weighted images became more uniform in the later phase. [file NBM-39-e70359-s001.docx]

Supplementary Table S1. Subject-level averaged SNR and SNR efficiency results.

| **Sequence type** | **SNR** | **SNR efficiency (s^-1/2^)** |
| --- | --- | --- |
| dMRA (proposed) | 13.61 ± 4.60 | 0.66 ± 0.22 |
| Perfusion (proposed) | 25.57 ± 12.13 | 1.25 ± 0.59 |
| dMRA (reference) | 11.90 ± 3.80 | 0.67 ± 0.21 |
| Perfusion (GRASE, reference) | 24.45 ± 10.89 | 1.36 ± 0.61 |


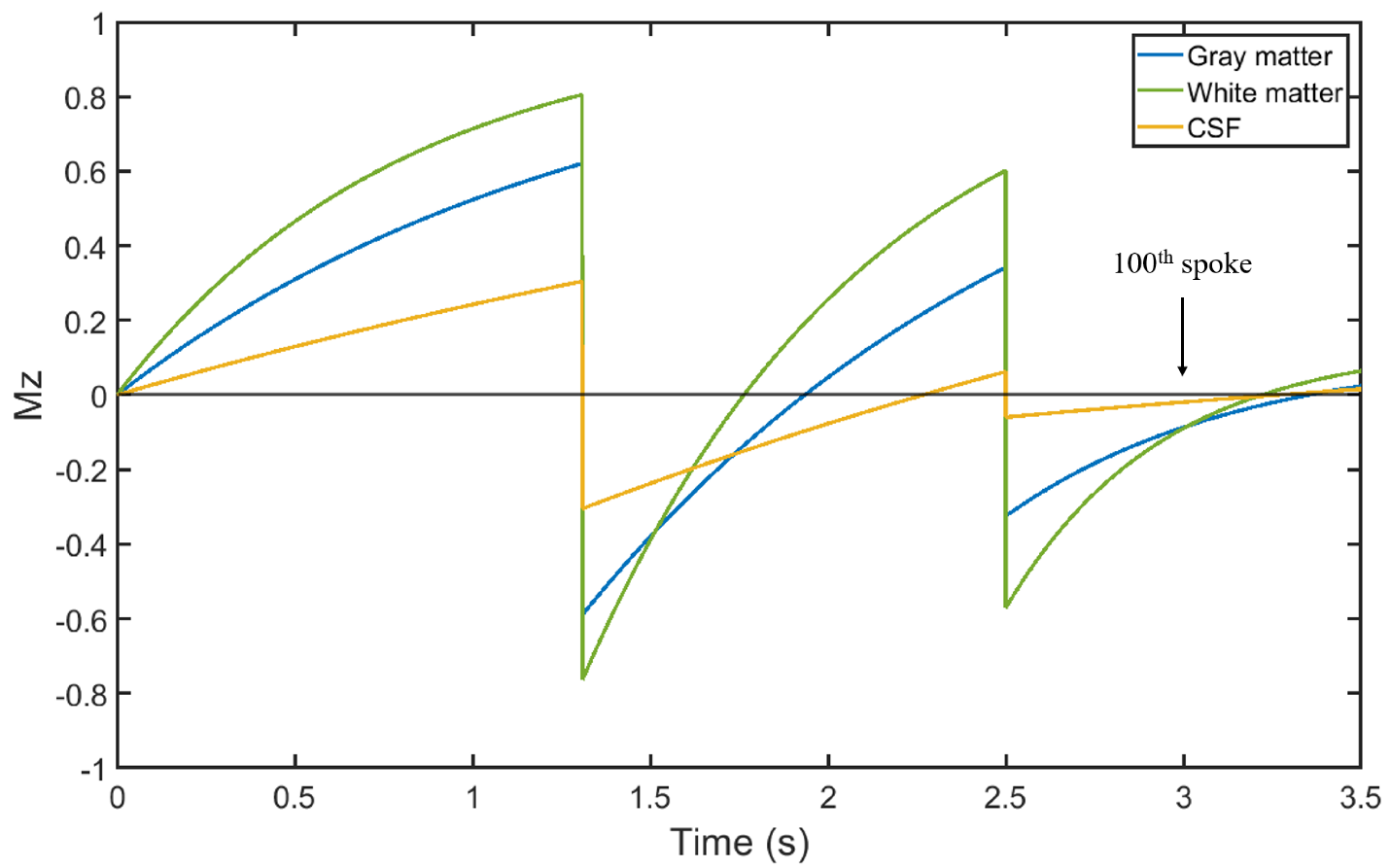


Supplementary Figure S1. Longitudinal magnetization evolution of tissue (gray matter, white matter, and cerebrospinal fluid) in the proposed technique, with the background suppression pulses applied at TI_1_ = 1309ms, TI_2_ = 2500ms, and considering the effect of readout.


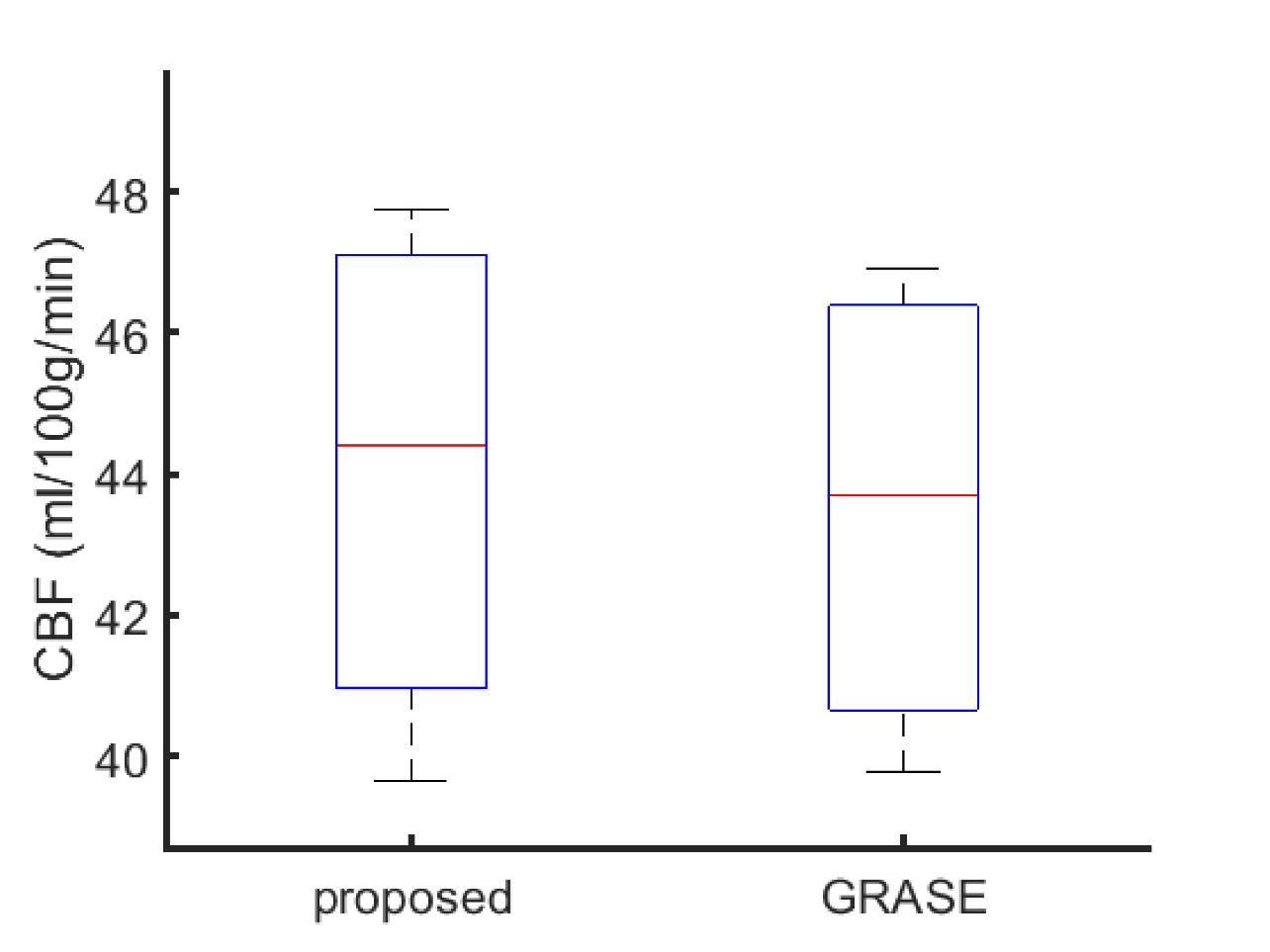


Supplementary Figure S2. Additional comparison of CBF between proposed technique and 3D GRASE reference using recommended parameters. No statistical difference was found in mean CBF values (p = 0.10, paired t-test, n = 4).


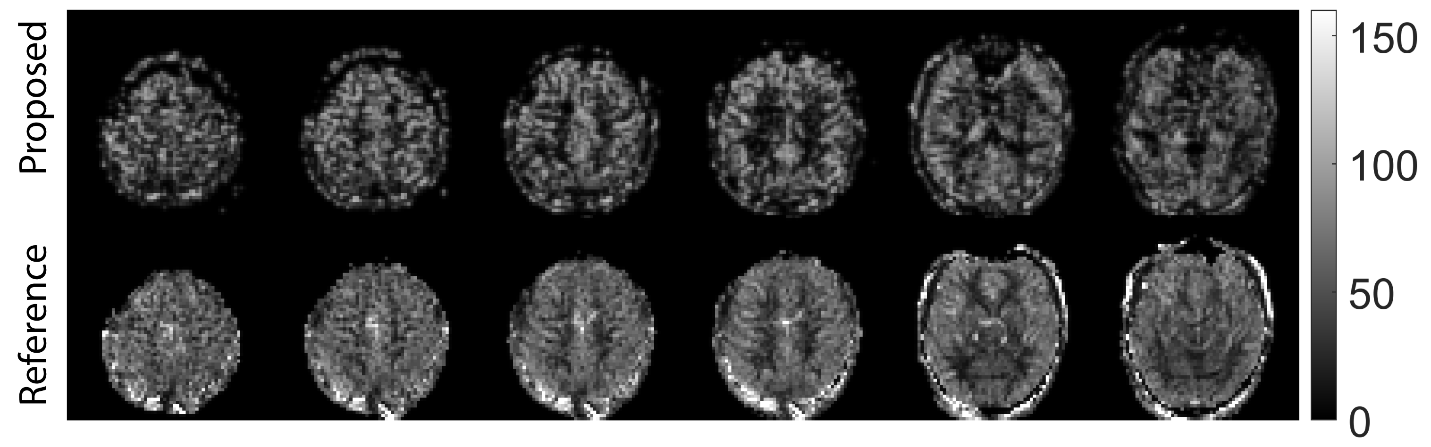


Supplementary Figure S3. Additional comparison of CBF maps between proposed technique and 3D GRASE reference using recommended parameters (unit: mL/100g/min). Some signal loss in frontal region from proposed technique was observed due to banding artifact from bSSFP readout.


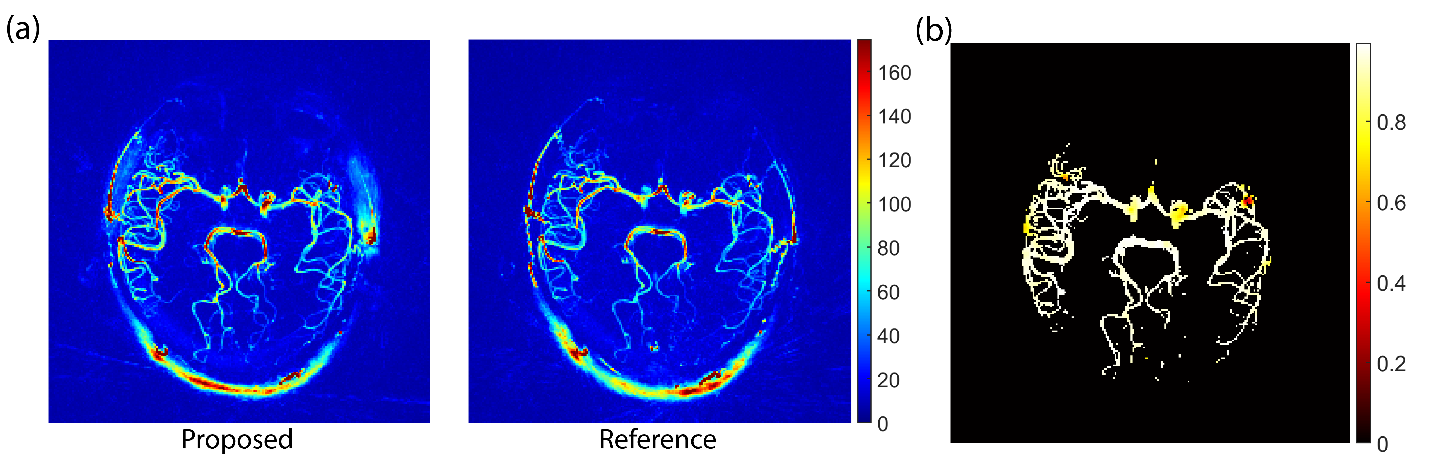


Supplementary Figure S4. (a) Representative SNR maps of transverse cMIP images from the proposed technique and the 4D MRA reference. (b) Corresponding SSIM map comparing transverse cMIP images from the proposed technique and the reference, with vascular regions highlighted. Similar SNR maps were achieved, and most vascular regions maintained high SSIM on the cMIP image.


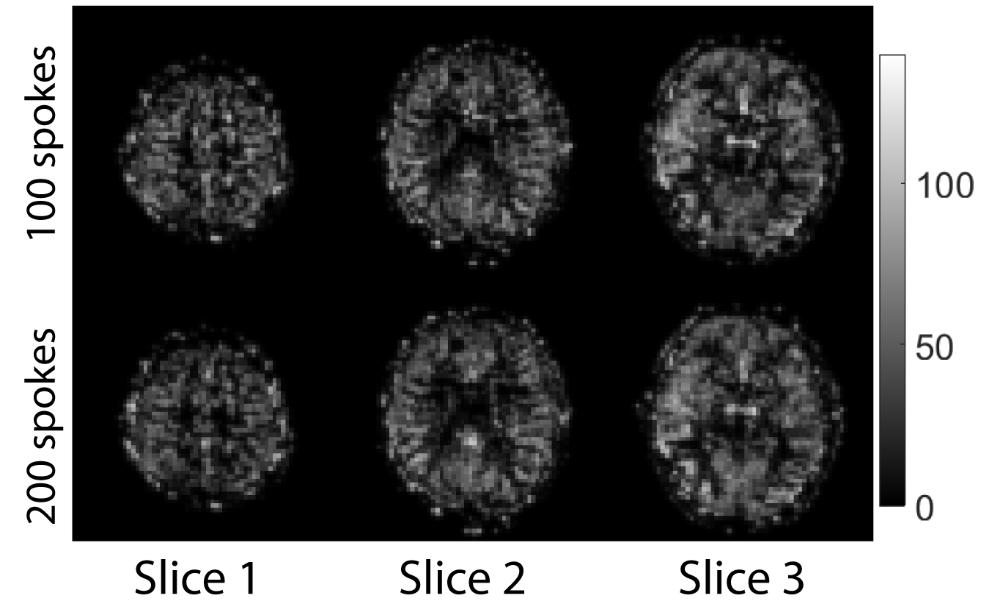


Supplementary Figure S5. CBF maps reconstructed using 100 and 200 radial spokes with the same effective PLD, respectively. The same quantification methodology was implemented for perfusion results with 100 radial spokes. Similar quality and CBF values were obtained when comparing the reconstruction with different radial spokes.


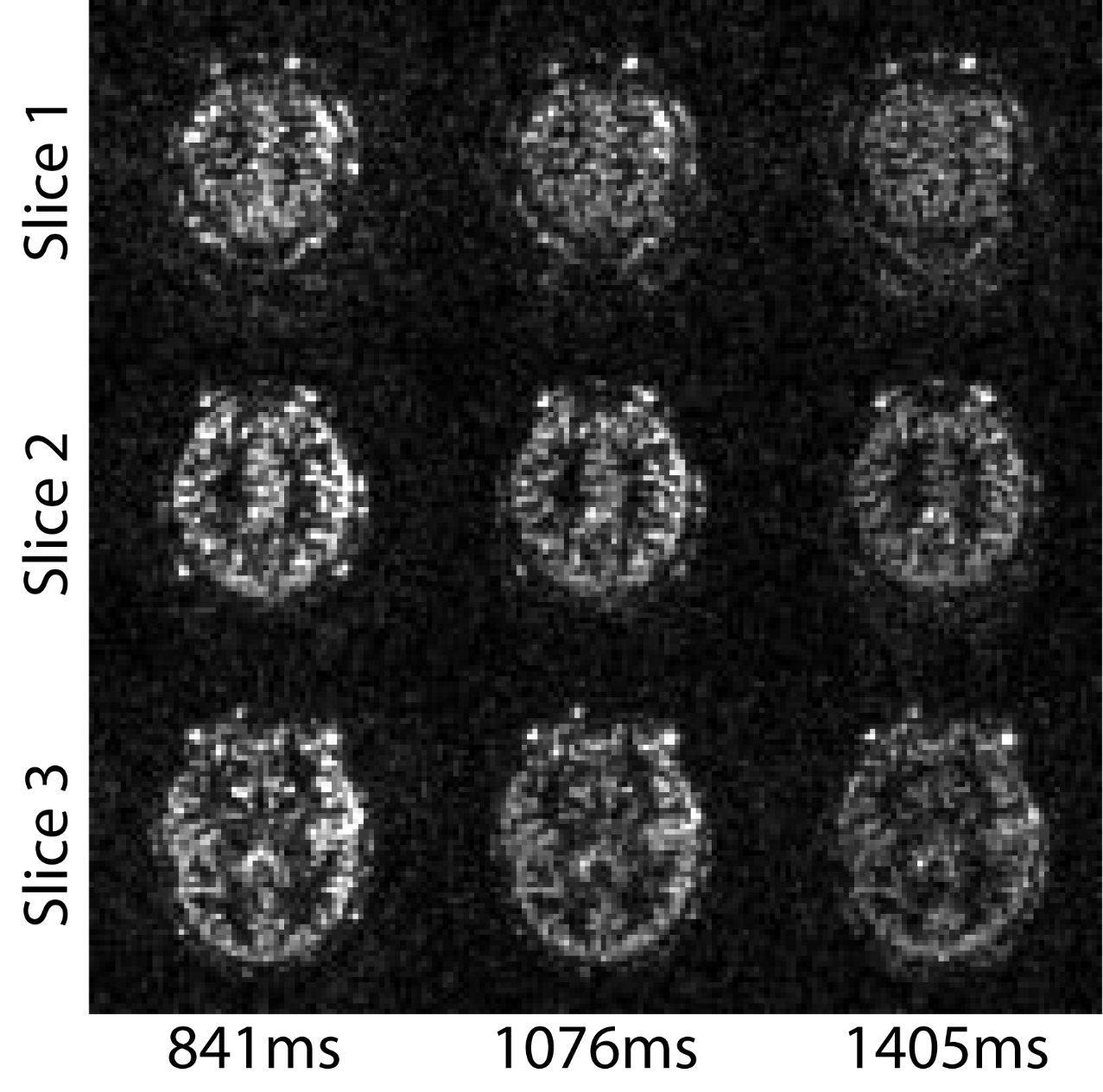


Supplementary Figure S6. Dynamic perfusion weighted images with different effective PLDs from three representative slices reconstructed with 60 radial spokes per frame. Local brightness was present in some distal vessels in the early phase, while the perfusion weighted images became more uniform in the later phase.
